# A Case of Fatal Acute Lung Injury after Balloon Valvuloplasty of Pulmonary Stenosis: Case Report and Review of Literature

**DOI:** 10.15171/jcvtr.2015.18

**Published:** 2015

**Authors:** Mohammad Ali Ostovan, Maliheh Kamali, Abdolali Zolghadrasli

**Affiliations:** ^1^ Department of Cardiology, School of Medicine, Shiraz University of Medical Sciences, Shiraz, Iran; ^2^ Shiraz Cardiovascular Research Center, Shiraz University of Medical Sciences, Shiraz, Iran

**Keywords:** Pulmonary Valve Stenosis, Percutaneous Pulmonary Valvuloplasty, Acute Lung Injury

## Abstract

A newly described immediate complication after percutaneous pulmonary valvuloplasty isacute lung injury. Here we report a case of fatal acute lung injury after pulmonary valvuloplasty.The patient was a 26-year-old woman, referred to a general hospital with the diagnosis of livercirrhosis. In her work-ups severe pulmonary stenosis was detected and so a decision was madeto relieve the valve stenosis. Despite the procedural success, the patient developed severe dyspneaand desaturation a few hours later and died within 3 days due to shock state. Although thedefinition, incidence or severity of acute lung injury after pulmonary balloon valvuloplasty is notyet clear, this is as far as we know the first mortality reported in literature. This presentation inour patient should prompt clinicians to consider a more aggressive approach at the first sight ofthis previously considered innocent complication.

## Introduction

Pulmonary valve stenosis is one of the most common forms of congenital heart diseases in the adults comprising about 5%-10% of all cases.^1-3^ Since its introduction in 1982, balloon valvuloplasty has become the method of choice to alleviate symptoms and improve pressure gradient across stenotic pulmonary valves.^1,3,4-7^ Despite the recent advances in technical success of this procedure, newer complications are coming into light.^1,3,6,8^ One such is acute lung injury in the first few hours after balloon valvuloplasty.^4,9^ Although this complication is reported to be common and usually mild, here we report a case of acute lung injury after balloon valvuloplasty which led to mortality in a 26 years old female adult.^10^

## Case Report

The patient was a 26-year-old woman who had first referred to a physician 4 months prior to admission due to facial edema and dyspnea on exertion which was attributed to upper respiratory infection. After 2 months she developed generalized edema, jaundice and pruritus. Because of abnormal liver tests, an abdominal sonography was done which was in favor of liver cirrhosis and so she was admitted to a tertiary care center for better management.

On admission she had blood pressure of 110/70 mm Hg, pulse rate of 80/min, respiratory rate of 20/min, temperature of 36.5°C orally. She was icteric, had pale conjunctiva, generalized edema but neurologically she had no signs of encephalopathy. Interestingly she had elevated jugular vein pressure and a harsh systolic murmur best heard in pulmonic area.

In order to assess the cardiac murmur, an echocardiography was done which demonstrated normal left ventricular size and function, but severe right ventricular hypertrophy and dilation with concomitant dysfunction and liver congestion. Further evaluation revealed severe valvular pulmonary stenosis associated with a gradient of 170-180 mm Hg which was later confirmed with a trans-esophageal echocardiography.

Upon this finding, the patient’s cirrhosis was attributed to pulmonary stenosis and because of favorable valvular morphology, percutaneous transvalvular pulmonary valvuloplasty was scheduled for the patient.

**Figure 1:**
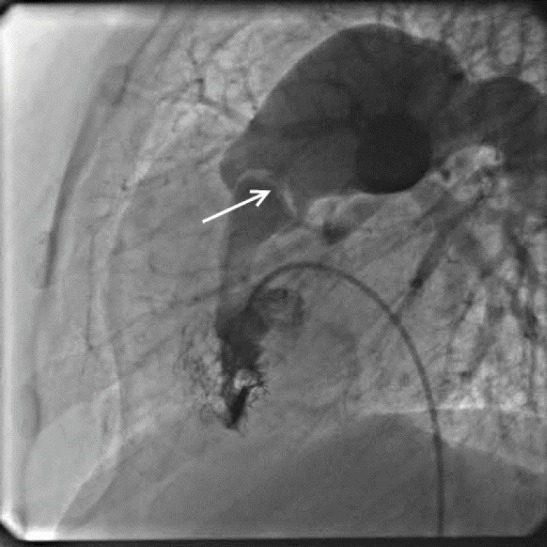
Right Ventricular Angiogram in Lateral View. The arrow points to the thick and domed pulmonary valve.

After enough hemostasis with fresh frozen plasma, she was transferred to the catheterization unit. Her angiogram showed thick and domed pulmonary valve and she had a peak to peak transvalvular gradient of 180 mm Hg (Figure 1). We used a 15 × 40 mm balloon initially to predilate the stenotic valve and then used a 20 × 45 mm balloon to fully dilate the pulmonary valve. The peak to peak transvalvular gradient dropped to 40 mm Hg after the procedure.

**Figure 2:**
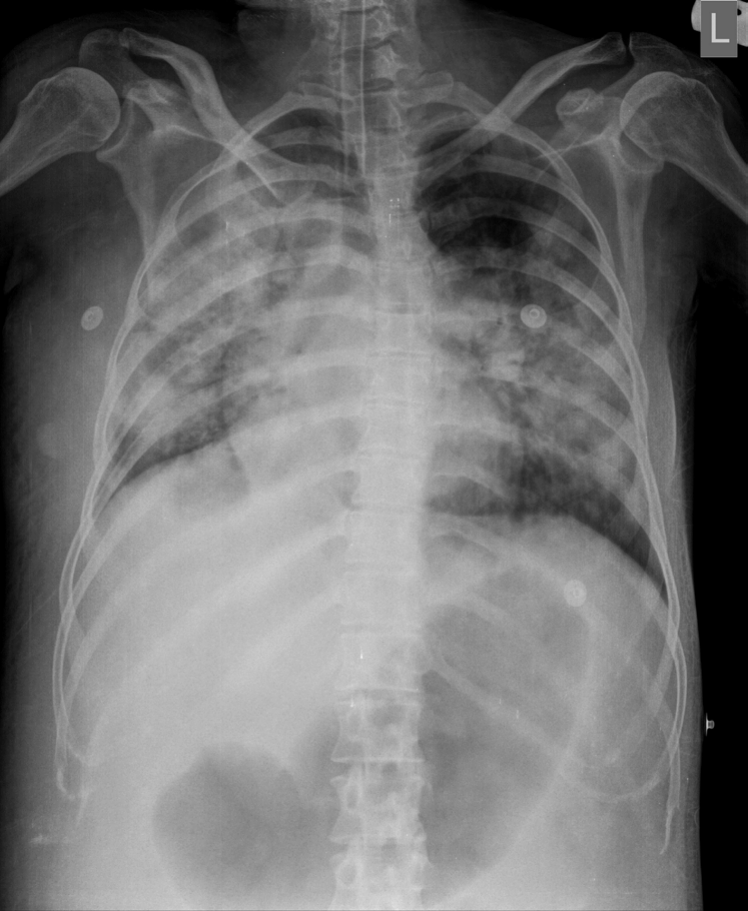
Portable Chest X-ray in the Patient a Few Hours After Balloon Valvuloplasty Which Shows Bilateral Diffuse Infiltration.

The patient was observed a few hours in the recovery room and was then transferred to the ward. After a few hours the patient gradually developed dyspnea and decreased oxygen saturation. Noninvasive oxygenation with facial mask was initiated. Her examination revealed blood pressure of 100/80 mm Hg, pulse rate of 128/min, respiratory rate of 28/min, cyanotic lips, diffuse bilateral fine rales over the lung fields and a harsh systolic murmur best heard in pulmonic area. Her blood gas analysis demonstrated mixed respiratory and metabolic acidosis and significant hypoxia and desaturation. A repeat echocardiography revealed trans-pulmonary valve gradient of 45 mm Hg associated with mild regurgitation without any other new findings. Portable chest X-ray revealed bilateral diffuse haziness (Figure 2). As she was unresponsive to non-invasive oxygenation with mask, she was intubated with endotracheal tube and transferred to the intensive care unit (ICU). In ICU she was ventilated with assist control mode with a rate of 12/min, tidal volume of 500 mL, partial oxygen fraction of 100% and PEEP of 5 mm Hg. Her 24-hour fluid intake was restricted to 1500 mL and she was given intravenous furosemide. Despite initial increase in arterial oxygen saturation, her condition remained critical with further deterioration in hemodynamics with decreased systolic blood pressure to 80 mm Hg and increased heart rate to 145/min. Her ICU course was further complicated by ventilator associated pneumothorax which was managed with chest tube insertion. Unfortunately despite all efforts, she died less than 3 days after being transferred to ICU due to shock state and severe oxygen desaturation.

## Discussion

Pulmonary balloon valvuloplasty has become the method of choice for the management of pulmonary stenosis, largely replacing surgical approach.^1,4-7,11^ This method has seen great advances in the past 2 decades, both because of technological advances and increased expertise.^4^ While early studies focused mainly on the success rate and immediate complications like pulmonary insufficiency, cardiac perforation, arrhythmia and tricuspid insufficiency, recent studies mainly focus on long-term outcomes and subtler complications.^3,4,7,11-13^ One such complications, first described in patients undergoing lung transplantation and congenital heart disease correction surgeries, is acute lung injury.^4,9,10,14^

Although acute lung injury was first described in 2001 in the subgroup of patients undergoing pulmonary balloon valvuloplasty, its incidence, degree and definition are not yet clear.^4,9^ Its incidence seems to be about 15%-22% after balloon valvuloplasty, 20%-40% after lung transplantation and up to 50% after congenital heart disease surgeries.^4,10,14^

The severity of symptoms also differ according to different reports; in one study all the patients suffering from acute lung injury after balloon valvuloplasty had to be transferred to cardiovascular intensive care unit while other reports consider it to be a self-limited condition.^4,10^ The context in which the injury happens seem to affect the severity of the clinical presentation; patients who developed acute lung injury after congenital heart disease operation seem to have less severe presentation than those after lung transplantion.^10^

The mechanism by which relief of high transvalvular gradient by balloon valvuloplasty produces acute lung injury is also a source of debate. Some advocate the theory of inflammation mediated reperfusion-ischemia injury as explained in lung transplantation patients, while others propose increase in end-diastolic volume of a noncompliant left ventricle.^4,10,14^ Another plausible mechanism is the acute increase in pulmonary blood flow after long-standing stenosis causing pulmonary edema or hemorrhage because of the inability of the microvasculature to restrict blood flow immediately and the subsequent increase in hydrostatic pressure.^9,10^ Whereas the increase in hydrostatic pressure seems to be the most prevalent and agreed-upon mechanism by which acute lung injury happens after pulmonary balloon valvuloplasty, the severity of the presentation in our patient leads us to hypothesize that inflammatory mediated reperfusion ischemic injury might have played a role

The prevention and management of this condition is also a point of interest and debate. Selection of a smaller balloon/annulus ratio while taking gradual steps in dilation of the valve is advocated by some authors.^9^ Currently intubation and mechanical ventilation seem to be a reasonable approach. There exists a report in which extracorporeal membrane oxygenation (ECMO) was applied successfully in a patient with acute lung injury after pulmonary valvuloplasty.^9^ In one study in patients undergoing lung transplantation, aprotonine – an agent which inhibits neutrophil extravasation- was successfully used to prevent acute lung injury.^14^ Considering the hypothesis of noncompliant left ventricle, the use of standard heart failure management may be another logical approach in such patients as utilized, albeit unsuccessfully, in our patient.

## Conclusion

We believe that the severe presentation of acute lung injury in this case, despite the utilization of staged approach in the relief of pulmonary stenosis, and the subsequent mortality, which as far as we know is the first reported in literature, should prompt other interventionists to consider a more aggressive approach at the first sight of this previously considered innocent complication. Close monitoring and early mechanical ventilation may probably save the patients. Application of aprotonine as a possible prophylactic agent and ECMO for the management of acute lung injury might worth a consideration to minimize the occurrence and mortality of this potentially life-threatening complication.

## Ethical Issues

The study was approval by our local Ethics Committee.

## Competing Interests

Authors declare no conflict of interest in this study.

## References

[1] Cheragh H, ul Hassan M, Hafizullah M, Gul AM (2009). Outcome of balloon pulmonic valvuloplasty with 18 months follow up. J Ayub Med Coll Abbottabad.

[2] Lip GY, Singh SP, de Giovanni J (1999). Percutaneous balloon valvuloplasty for congenital pulmonary valve stenosis in adults. Clin Cardiol.

[3] Lin SH, Hwang JJ, Hsu KL (2004). Balloon pulmonary valvuloplasty in adults with congenital valvular pulmonary stenosis. Acta Cardio Sin.

[4] Yacouby S, Meador M, Mossad E (2014). Lung reperfusion injury in patients after balloon angioplasty for pulmonary artery stenosis. J Cardiothorac Vasc Anesth.

[5] Fedderly RT, Lloyd TR, Mendelsohn AM, Beekman RH (1995). Determinants of successful balloon valvotomy in infants with critical pulmonary stenosis or membranous pulmonary atresia with intact ventricular septum. J Am Coll Cardiol.

[6] Behjati-Ardakani M, Forouzannia SK, Abdollahi MH, Sarebanhassanabadi M (2013). Immediate, short, intermediate and long-term results of balloon valvuloplasty in congenital pulmonary valve stenosis. Acta Med Iran.

[7] Weryński P, Rudziński A, Król-Jawień W, Kuźma J (2009). Percutaneous balloon valvuloplasty for the treatment of pulmonary valve stenosis in children - a single centre experience. Kardiol Pol.

[8] Kim do H, Park SJ, Jung JW, Kim NK, Choi JY (2013). The Comparison between the Echocardiographic Data to the Cardiac Catheterization Data on the Diagnosis, Treatment, and Follow-Up in Patients Diagnosed as Pulmonary Valve Stenosis. J Cardiovasc Ultrasound.

[9] Cheng HI, Lee PC, Hwang B, Meng CC (2009). Acute pulmonary reperfusion hemorrhage: a rare complication after oversized percutaneous balloon valvuloplasty for pulmonary valve stenosis. J Chin Med Assoc.

[10] Asija R, Roth SJ, Hanley FL (2014). Reperfusion pulmonary edema in children with tetralogy of Fallot, pulmonary atresia, and major aortopulmonary collateral arteries undergoing unifocalization procedures: A pilot study examining potential pathophysiologic mechanisms and clinical significance. J Thorac Cardiovasc Surg.

[11] Kaul UA, Singh B, Tyagi S, Bhargava M, Arora R, Khalilullah M (1993). Long-term results after balloon pulmonary valvuloplasty in adults. Am Heart J.

[12] Radtke W, Keane JF, Fellows KE, Lang P, Lock JE (1986). Percutaneous balloon valvotomy of congenital pulmonary stenosis using oversized balloons. J Am Coll Cardiol.

[13] Rocchini AP, Kveselis DA, Crowley D, Dick M, Rosenthal A (1984). Percutaneous balloon valvuloplasty for treatment of congenital pulmonary valvular stenosis in children. J Am Coll Cardiol.

[14] Bittner HB, Binner C, Dahlberg P, Mohr FW (2007 Mar). Reducing ischemia-reperfusion injury in clinical lung transplantation. Transplant Proc.

